# The Local Topological Reconfiguration in the Brain Network After Targeted Hub Dysfunction Attacks in Patients With Juvenile Myoclonic Epilepsy

**DOI:** 10.3389/fnins.2022.864040

**Published:** 2022-04-14

**Authors:** Ming Ke, Huimin Li, Guangyao Liu

**Affiliations:** ^1^School of Computer and Communication, Lanzhou University of Technology, Lanzhou, China; ^2^Department of Magnetic Resonance, Lanzhou University Second Hospital, Lanzhou, China

**Keywords:** rich-club, eigenvector centrality, Crucitti-Latora-Marchiori, resting-state network, juvenile myoclonic epilepsy

## Abstract

The central brain regions of brain networks have been extensively studied in terms of their roles in various diseases. This study provides a direct measure of the brain’s responses to targeted attacks on central regions, revealing the critical role these regions play in patients with juvenile myoclonic epilepsy (JME). The resting-state data of 37 patients with JME and 37 healthy subjects were collected, and brain functional networks were constructed for the two groups of data according to their Pearson correlation coefficients. The left middle cingulate gyrus was defined as the central brain region by the eigenvector centrality algorithm and was attacked by the CLM sequential failure model. The rich-club connection differences between the patients with JME and healthy controls before and after the attacks were compared according to graph theory indices and the number of rich-club connections. We found that the numbers of rich connections in the brain networks of the healthy control group and the group of patients with JME were significantly reduced [*p* < 0.05, false discovery rate (FDR) correction] before the CLM sequential failure attacks, and no significant differences were observed between the feeder connections and local connections. In the healthy control group, significant rich connection differences were obtained (*p* < 0.01, FDR correction), and no statistically significant differences were observed regarding the feeder connections and local connections in the brain network before and after CLM failure attacks on the central brain region. No significant differences were obtained between the rich connections, feeder connections, and local connections in patients with JME before and after CLM successive failure attacks on the central brain area. The rich connections, feeder connections, and local connections were not significantly different in the brain networks of the healthy control group and the group of patients with JME after CLM successive failure attacks on the central brain region. We concluded that the damage to the left middle cingulate gyrus is closely linked to various brain disorders, suggesting that this region is of great importance for understanding the pathophysiological basis of myoclonic seizures in patients with JME.

## Introduction

Juvenile myoclonic epilepsy (JME) is a highly prevalent genetic generalized epilepsy syndrome, which accounts for up to 10% of all epilepsy cases (Camfield et al., 2013; Scheffer et al., 2017). JME is a common type of idiopathic generalized epilepsy that occurs mostly in people aged 12–18 years, and it accounts for 8–10% of the prevalence in the epileptic population. The clinical manifestations of JME are mainly myoclonic seizures, disorientation seizures, generalized tonic-clonic seizures, cognitive impairment, and motor impairment (Wolf et al., 2015). At present, the complex network method of functional magnetic resonance imaging (fMRI)-based data analysis has been widely used in the study of neuropsychiatric diseases. JME studies conducted with complex networks based on graph theory suggest that JME episodes likely originate in cortical networks associated with movement (Krauss, 2011). The associations between identified discharge-affecting networks and the relationships among resting-state networks might be influenced by aspects of epilepsy in patients with JME (Dong et al., 2016). Brain network studies have pointed to the fact that alterations in brain network organization are closely associated with brain disorders (Liu et al., 2008; Bassett and Bullmore, 2009; Fornito et al., 2015). Our existing CLM sequential failure model for adolescent myoclonic epilepsy revealed that the left middle frontal lobe might be a potential focal area regarding the initiation of generalized tonic-clonic seizures (Ke et al., 2019).

However, there is currently no clear understanding of the internal causes of these large-scale network changes. A key hypothesis was that central brain region dysfunction was likely to be associated with disease (van den Heuvel et al., 2013). A highly central and interconnected organization was presented for the brain network, which played a crucial role in the integration process of the brain, forming the central backbone of global brain communication. The hub region at the center of the brain network topology is the center of information integration between different subsystems of the human brain, and it is capable of collaboratively integrating multisensory information (Zamora-Lopez et al., 2010; Zuo et al., 2012). Central nodes play pivotal roles in the overall architectures of functional brain networks (Sporns et al., 2007; Sporns, 2011; van den Heuvel and Sporns, 2011). These regions of information transmission form highly aggregated brain communication hubs that make important contributions to cross-regional information transmission (van den Heuvel et al., 2013). It is known from past studies of diseases that abnormalities in the central brain regions of patients with schizophrenia lead to brain network topology reorganization, resulting in reduced connectivity within the brain network (van den Heuvel et al., 2013). Moreover, a neuroimaging study reported that the presence of abnormal brain network topologies in patients with JME increased the importance of the motor-related cortex in functional brain networks, which implied a reorganization of the central network nodes in the patients with JME. The changed topologies of the brain networks in the patients with JME caused connectivity to decrease within these brain networks (Ray et al., 2014). From the above description of the central region, we know that the central region plays an important role in brain network topology and information transmission. Once abnormalities occur in the central region, the brain network is greatly affected. Therefore, it is crucial to comprehensively understand the causal relationship between the central nodes of the brain network and the network topology.

We used the CLM sequential failure model to assess the role played by lesions of the central brain regions in brain networks. The CLM sequential failure model is a submodel of the load-capacity model, also known as the node-edge hybrid dynamic model, which does not separate the two factors of nodes and edges but rather considers the importance of both and the role they play in load distribution. Unlike previous approaches to node failure, the CLM model does not remove the node directly but, instead, reduces the efficiency of the edges associated with the node (Crucitti et al., 2004). Kinney et al. (2005) applied the CLM sequential failure model to a North American power network and found that the overall performance of the network degrades relative to the normal state when attacking the highest-loaded generation or transmission nodes. Using the CLM sequential failure model to simulate seizures and their dynamic propagation process in patients with epilepsy, our laboratory found that the left middle frontal lobe may be a potential focal area in the onset of generalized tonic-clonic seizures (Ke et al., 2019). CLM successive failures are already present in the application of the model to complex networks. The model is an important inspiration for our study.

This research adopted the eigenvector centrality (EC) algorithm by choosing the left cingulate gyrus regions of the brain network as central brain regions. The CLM sequential failure model was used to attack the central brain network regions of the healthy control group and the JME group. To study the influence of the central node abnormalities on brain networks, as well as the relationships between these abnormalities and the causes of disease, a rich-club connection analysis was performed on the brain networks of the two groups before and after the attacks. The results showed that the abnormal central nodes in the brain networks of patients with JME led to brain network topology reorganization, affected the functional connections of the brain networks, and obstructed functional information transmission. This is of great significance for understanding the pathophysiological mechanism of JME and indicates that these central brain regions are central brain regions of adolescent clonic epilepsy.

## Materials and Methods

### Participants

Resting-state fMRI data were collected from 37 patients with JME (with average illness duration of 4.03 years) admitted to the Epilepsy Center of Lanzhou University Second Hospital. The patients were diagnosed with JME according to the criteria for epilepsy classification contained in the International League Against Epilepsy (ILAE) guidelines of 2001. Patients were excluded if they had any of the following characteristics: (1) a history of antiepileptic medication intake, (2) other neurological or psychiatric illness, (3) other developmental disabilities, such as autism and intellectual impairment, and (4) an acute physical illness that would affect the scanning. To evaluate the severity of the epilepsy cases, each patient was required to complete the National Hospital Seizure Severity Scale (NHS3) before the MRI examination. This scale contains six seizure-related factors and produces a total score from 1 to 23. The patients included 21 males and 16 females with an average age of 17.757 ± 5.33 years. No structural abnormalities were found during the routine MRI examinations; the electroencephalography (EEG) results obtained during seizures exhibited extensive multispinous slow waves or compound spinous slow waves at 4∼6 Hz; and none of the patients had received formal treatment. At the same time, the fMRI and 3DT1 images of 37 normal volunteers, including 13 males and 24 females with an average age of 20.081 ± 4.723 years, were selected. The subjects in both groups were right-handed. No significant differences in age, handedness, or sex were observed between the two groups after conducting intragroup paired false discovery rate (FDR) correction (*p* > 0.05). The normal volunteers were recruited through advertising and excluded before excluding those with acute physical illness, substance abuse or dependence, a history of loss of consciousness due to craniocerebral injury, and neurological or psychiatric disorders. This study was approved by the Ethics Committee of the Second Hospital of Lanzhou University, and written informed consent was obtained from each subject or his or her legal guardian after the experimental protocol. Subject information is shown in Table 1.

**TABLE 1:** Subject information.

Variables	JME (*n* = 37)	HC (*n* = 37)
Sex	21/16	13/24
Mean age (year)	17.757 ± 5.33	20.081 ± 4.723
Mean disease duration (months)	46.919 ± 10.627	0
Handedness (right/left)	37/0	37/0
Mean score of the NHS3	9.054 ± 3.778	0
Mean first time of onset	3.185 ± 2.661	0

*NHS3, National Hospital Seizure Severity Scale.*

### Data Acquisition

All data were collected by a Siemens Verio 3.0 T MR scanner. The data collection process required each subject to lie supine, fix his or her head, close his or her eyes, plug his or her ears, and try not to perform specific thinking. The fMRI data were collected *via* a gradient-echo echo-planar imaging (GRE-EPI) sequence. The specific parameters were as follows: the repetition time (RT) = 2,000 ms, echo time (TE) = 30 ms, slice thickness = 4 mm, slice gap = 0.40 mm, number of layers = 33 layers, field of view (FOV) = 240 mm × 240 mm, in-plane matrix resolution = 64 × 64, and flip angle (FA) = 90°, and 200 time points were collected in total. T1-weighted images were acquired by a 3D magnetization-prepared rapid gradient echo sequence (3D MP-RGE). The specific parameters were as follows: the repetition time (TR) = 1,900 ms, TE = 30 ms, slice thickness = 0.9 mm, FOV = 256 mm × 230 mm, matrix = 256 × 256, FA = 90°.

### Data Preprocessing

We implemented the data preparation process in MATLAB version 2013a, which ran on the Windows 10 operating system. The graph theoretical network analysis toolbox (GRETNA)^1^ (Wang et al., 2015), which ran on MATLAB, was also used to preprocess the data. The main steps of preprocessing included (1) formatted conversion of the DICOM data (conversion of the two-dimensional images in the DICOM format into three-dimensional images in the NIFTI format); (2) removal of the first few time points: the first 10 time points were selected to eliminate data instability caused by reasons such as the starting of the mcassociations between identifiedahine; (3) slice-timing correction, in which all scan time points were corrected to the same reference point. The interval time is 2 s, and there are 33 time layers in total. Using interlayer scanning, the scanning order alternated in the positive direction starting with odd-numbered slices (i.e., 1, 3, 5,… 2, 4, 6, …); (4) head movement correction if the translation was greater than 1 mm, and the rotation was greater than 1 degree; (5) within-subject coregistration of the T1 image to a mean functional image; (6) spatial normalization: each scan was stretched, compressed, and coiled to make the scanned brain consistent with a standard brain template; (7) removal of linear drift: removing linear trends that arose from warming or subjects not adapting due to the functioning of the machine; (8) low-pass filtering: the range of low-pass filtering was 0.01–0.08 Hz; and (9) regressing out of the covariates: for the fMRI datasets, several nuisance signals were typically removed from each voxel’s time series to reduce the effects of non-neuronal fluctuations, including head motion profiles, cerebrospinal fluid signals, white matter signals, and global signals.

### Brain Network Construction

In graph theory, the brain network refers to a collection of nodes (brain areas) and edges (connections). The automated anatomical labeling (AAL) brain atlas was employed to define the regions of interest (ROIs), and the ROI function was employed to compute the functional connectivities between the 90 nodes. To construct the brain functional network, the response sequence of each brain region of each subject was extracted over time. The Pearson correlation coefficient was used to obtain the correlation coefficient matrix over time. The expression is as follows:


(1) $$r\,\left(x_{n},y_{n}\right)=\frac{\sum \left(x_{n}-\overset{\_}{x}\right)\,\left(y_{n}-\overset{\_}{y}\right)}{\sqrt{\sum {\left(x_{n}-\overset{\_}{x}\right)}^{2}\,\sum {\left(y_{n}-\overset{\_}{y}\right)}^{2}}}$$

*x*_*n*_ is the time series of the n-th brain region. $\bar{x}$ is the mean value across all scan layers in the brain region. *y*_*n*_ and $\bar{y}$ are the time series and mean value of another brain region. When the correlation coefficient *r*(*x*_*n*_,*y*_*n*_) was greater than a given threshold value *T*m**, it was considered that there was a functional connection between *x*_*n*_ and *y*_*n*_; i.e., an edge connection existed between them.

**FIGURE 1:**
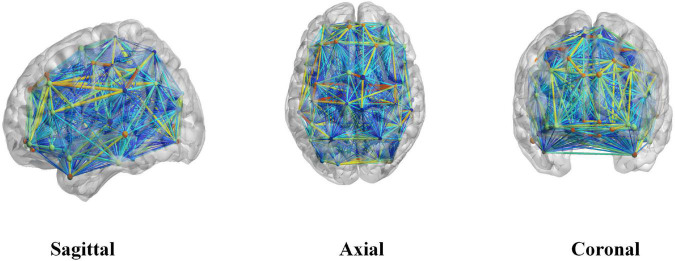
A brain network connectivity map generated using the BrainNet Viewer toolbox.

In this experiment, we performed Fisher’s z-transformation on the brain functional connectivity. First, binarization was performed on the brain network; when the connection value between two brain regions was greater than the threshold value, they were considered to have an edge, whose value was set to 1; otherwise, the edge value was set to 0. In this experiment, we chose 0.3 as the threshold value in constructing the binary brain network; at this value, the network density is between 10 and 50%, and the average network degree < *k* > is greater than two times the natural logarithm of the number of network nodes *N*. In this case, this also yielded no isolated nodes in the brain network, and so the entire network has connectivity. These are the two rules to follow when building a brain network, and choosing this value for the threshold ensures that our brain network satisfies these two rules. Figure 1 shows the connectivity graph of the brain network generated using the BrainNet Viewer toolbox.

### Eigenvector Centrality

Eigenvector centrality is a measure of the importance of a node and depends on both the number (i.e., the degree of the node) and the importance of its neighbor nodes. The EC of a node was proportional to the sum of the centrality scores of its neighbor nodes. The centrality of the eigenvector vectors emphasized the surrounding environment (the number and quality of node neighbors), which, in essence, meant that a node’s score was the sum of its neighbors’ scores. A node could increase its importance by connecting to many other important nodes. A node with a high score could be connected to either a large number of ordinary nodes or a small number of other high-scoring nodes. From the perspective of transmission, the centrality of eigenvectors was suitable for describing the long-term influence of nodes. For example, during the spread of a disease or spread, a larger EC score indicates that the corresponding node is more likely to be closer to the source of infection and is a key node that needs to be prevented (Bonacich and Lloyd, 2001).

The expression is as follows: if *x*_*i*_ is an important measure of node *I*, then:


(2) $$\mathrm{EC}\,\left(i\right)=x_{i}=c\,\sum_{j=1}^{n}a_{\mathrm{ij}}\,x_{j}$$

where, *c* is a proportionality constant, *x* = (*x*_1_, *x*_2_, *x*_3_,…, *x*_*n*_)*^T^*, and when the steady state is reached after multiple iterations, it can be written as the following expression:


(3) x=c⁢A⁢x

This means that *x* was the eigenvector corresponding to the eigenvalue *c* of Matrix *A* minus 1. The basic method for calculating the vector *x* was to give an initial value *x*(0) and then use the following iterative algorithm:


(4) x⁢(t)=c⁢A⁢x⁢(t-1),t= 1,2⁢…

Then, it was normalized: x′(*t*) = *x*′(*t*−1). During each iteration step, if *x* was divided by λ, a convergent non-zero solution *x* = *Ax*/λ could be obtained for this equation, where λ is the principal eigenvalue of the binarized brain network adjacency Matrix *A*, i.e., the constant *c* = 1/λ.

From the perspective of the method itself, EC identifies the importance of a node (within network) by considering the quality of its connections (not only how many connections it has but also whether the connections are formed with important nodes). An increasingly popular centrality metric, the EC is unique in that it considers the centrality of immediate neighbors when computing the centrality of a node. Mathematically, EC is a positive multiple of the sum of the adjacent centralities and is based on the philosophy that a node is more central if its neighbors are also highly central. The novelty of the approach is that it assigns an “importance” score to each feature by considering all other features mapped as nodes on the graph, bypassing the combinatorial problem in a methodologically sound fashion. Indeed, EC differs from other measurements (e.g., degree centrality) since a node (feature) with many links does not necessarily have a high EC because not all nodes are equivalent, some are more relevant than others, and, reasonably, endorsements from important nodes count more. The analysis presented by Lohmann et al., shows that feature vector centrality is an effective computational tool for capturing intrinsic neural structure at the voxel level. Therefore, the method is informative in measuring the importance of nodes.

We used the principle of EC to obtain the EC value of each node and selected the central nodes of the brain network according to the ranked values. This node represents the attack node in subsequent CLM failure attacks.

### Successive Failure of CLM

The CLM model not only separated the two factors of nodes and edges but also considered the importance of nodes and edges in terms of load allocation by studying their joint effects. The CLM model did not remove nodes directly but instead reduced the efficiency of the edges associated with the nodes. The principle was that the information passing through a faulty node would choose other alternative transmission paths.

From the point of view of graph theory, assuming that the network was an undirected weighted graph *G*, the transmission of network information between any two points was always conducted along the shortest path, namely, the optimal path with respect to transmit efficiency. The value of the edge between nodes *i* and *j* was *e*_*ij*_, which was expressed by the reciprocal of the shortest path length *L*_*ij*_ between the nodes (when there was no path between nodes *i* and *j*, the reciprocal of the shortest path length value *L*_*ij*_ was 0), which represented the transmission efficiency between nodes *i* and *j* at time *t*; the associated formula is:


(5) ε⁢ij=1/d⁢ij

The construction of this model was divided into two parts: the load distribution without node failure and the load distribution after node failure.

(1) Before a node failure occurred, *L*_*i*_(*t*) was defined to represent the load of node *i* at time *t.* Additionally, *L*_*i*_(*t*) represented the number of paths passing through the shortest distance from node *i* at time *t*. When *t* was 0, the initial load was *L*_*i*_(0). *C*_*i*_ was the capacity of node *i*, and its calculation expression was:


(6) $$C_{i}=a\cdot L_{i}\left(0\right)\left(a\geq 1\right)$$

where, *C*_*i*_ represents the capacity coefficient, which represents the ability of nodes to handle the load and transmit information.

(2) When a failure occurred, an attack on a node inevitably changed the shortest paths between nodes, resulting in a redistribution of the total load. The load redistribution among the nodes might have led to the overload of other nodes, which, in turn, led to a new round of load redistribution, leading to successive failures. In this model, when a node was overloaded, the load allocated to this point was gradually reduced by reducing the transmission efficiency of the edge connected to it. When the load was less than the capacity, the node worked normally again. The internode efficiency iteration formula at time *t* is:


(7) $$e_{i\,j}\,\left(t+1\right)=\left\{ \begin{array}{c} e_{i\,j}\,\left(0\right)\cdot \frac{C_{i}}{L_{i}\,\left(t\right)},L_{i}\,\left(t\right)>C_{i}\\ e_{i\,j}\,\left(0\right),L_{i}\,\left(t\right)\leq C_{i} \end{array}\right.$$

In this study, we focused on the calculation of the brain network matrices before and after attacks. The successive brain network fault matrix removed all edges related to the central node (when the edge value of the node connected to the central node was reduced to 0; otherwise, the value remained unchanged). The edge connection values of the remaining nodes did not change. The brain networks before and after successive failures were taken as the input values of the rich-club structure, and the rich club was used to analyze the results.

To study the changes in brain network topology before and after successive fault attacks, we used the graph theory indices of complex networks to study the difference in brain network information transmission before and after successive CLM fault attacks. The topological changes in the brain networks before and after successive failures were measured by rich-club connections.

### Graph Theory Indicators

To study the role of the cingulate gyrus in disease, we adopted a CLM sequential failure model to attack the cingulate gyrus and simulate the process of cingulate gyrus damage. Then, we used the graph theory indices of complex networks to analyze the difference between the brain networks after successive CLM failure attacks occurred. In this experiment, we used the clustering coefficient and global efficiency as two graph theory indicators for study and analysis.

#### Clustering Coefficient

The clustering coefficient was an important parameter that was used to describe the nodes’ tightness in the network. For a node *i* with a degree value of *k*_*i*_, its clustering coefficient *c*_*i*_ was defined as the ratio of the actual number of edges between the *k*_*i*_ nodes connected to node *i* and the possible number of edges between these nodes *e*_*i*_, namely:


(8) $$c_{i}=\frac{2\,e_{i}}{k_{i}\,\left(k_{i}-1\right)}$$

The clustering coefficient *C* of the entire network was defined as the mean clustering coefficient *c*_*i*_ across all nodes in the network, namely:


(9) $$C=\frac{1}{N}\,\sum_{i=1}^{N}c_{i}$$

#### Global Efficiency

When isolated nodes were contained in the network, the shortest path length of the network was infinite. To avoid this problem, someone put forward the concept of efficiency. In-network efficiency (the global efficiency), which measured the global information transmission ability, was *E*_*glob*_.

The global efficiency was defined as the mean of the reciprocals of the shortest path lengths between all pairs of nodes in the network, i.e.,


(10) $$E\,g\,l\,o\,b=\frac{1}{N\,\left(N-1\right)}\,\sum_{i,j\in V,i\neq j}\frac{1}{l_{i\,j}}$$

where, *N* represents the number of nodes in the brain network and *l*_*ij*_ represents the length of the shortest path between nodes *i* and *j* in the subgraph.

### Rich Club

A rich club is defined as a group of nodes in a random network whose connectivity level exceeds the expected connectivity level. Here, the degree value *k* of each node in the functional network of the healthy control group was first calculated. The detection process involved the following steps. (1) We discarded all nodes in the network whose degrees were less than *k*. (2) Then, we calculated the ratio of the connections between the remaining nodes and the total number of possible connections when the network was in a fully connected state. (3) For each *k*, the normalized rich-club coefficient was divided by the average rich-club coefficient of 100 random networks (Colizza et al., 2006).

The normalized rich-club coefficient *φnorm*(*k*) was the ratio of the rich-club coefficient φ(*k*) to *φrandom*(*k*), where, *φrandom*(*k*) represents 100 random brain networks. The expression is as follows:


(11) $$\phi \,n\,o\,r\,m\,\left(k\right)=\frac{\phi \,\left(k\right)}{\phi \,r\,a\,n\,d\,o\,m\,\left(k\right)}$$

where, φ(*k*) is expressed as:


(12) $$\phi \,\left(k\right)=\frac{2\,E_{>k}}{N_{>k}\,\left(N_{>k}-1\right)}$$

In the formula, *E*_>*k*_ represents the number of connections between nodes whose degrees were greater than *k* in the network, and the denominator represents the total number of possible connections between the nodes when the degree was set to “full connection.” *N_>*k*_* denotes the number of nodes whose degree was greater than *k*. *E*_>*k*_ took the node degree *K* as the threshold and constantly adjusted *k* to remove the nodes whose node degrees were less than or equal to *K* from the network. In this experiment, the value of *K* ranged from 1 to 89, and each value was utilized one time to obtain a corresponding normalized rich-club coefficient. Through the three steps of the rich-club test, this paper found that the normalized rich-club coefficients of the two groups of brain networks were greater than 1 in a certain range, which was consistent with the results obtained by existing studies (Li et al., 2016). The normalized rich-club coefficients were obtained through a one-sample *t*-test. It was proved that the brain functional networks of both the healthy control group and the JME group had rich-club characteristics, which could be used for subsequent rich-club studies.

Rich-club connections refer to the three types of connections that can be formed according to the utilized node selection standard: (1) rich connections, i.e., connections between rich-club nodes; (2) feeder connections, i.e., connections between rich-club and non-rich-club nodes; and (3) local connections, i.e., connections between non-rich-club nodes.

In this study, the brain networks of 37 healthy controls were first calculated, and the average brain network of the 37 healthy controls was constructed. On this basis, the degree value of each node in the average brain network was obtained. The node selection criteria included that the top 10% of nodes among the 90 ranked nodes in terms of their degrees were selected as “rich nodes,” and the rest were defined as “non-rich nodes” (Yan et al., 2018). According to the node selection criteria, nine regions in the brain network satisfied the “rich node” selection criteria, including the left middle cingulate gyrus, the left anterior central gyrus, the right anterior central gyrus, the left precuneus, the left dorsolateral superior frontal gyrus, the right dorsolateral superior frontal gyrus, the left supplementary motor area, the left parahippocampal gyrus, and the left middle frontal gyrus. Therefore, these nine brain regions were defined as “rich nodes,” while the other 81 brain regions were defined as “non-rich nodes.” The rich nodes and non-rich nodes were used to classify the three kinds of connections. To ensure the reliability of the control experiment, the same “rich nodes” and “non-rich nodes” were selected from the healthy control group and the group of patients JME for the division of the three kinds of connections instead of selecting the “rich nodes” from the healthy control group. In the group of patients with JME, the “rich nodes” of the brain network for the patients with JME were selected according to the node selection criteria, and then the three connection values were calculated. The rich connection value was calculated as the sum of the weights of the connections between all rich nodes in the brain network. The feeder connection value was calculated as the sum of the weights of the connections existing between rich nodes and non-rich nodes. The local connection value was the sum of the weights of all connections that existed between non-rich nodes.

## Results

### The Eigenvector Centrality Results

The EC results for the top 10% of the node degrees in the 90 brain regions are shown in Table 2. The left side shows the top nine brain regions among the 90 brain regions in terms of EC, and the right side shows the top nine EC values among the 90 brain regions as calculated by the EC algorithm. The EC value of the left middle cingulate gyrus was the highest in Table 2.

**TABLE 2:** Eigenvector centrality results.

Brain area	Eigenvector centrality results
Left middle cingulate gyrus	0.1650
Right middle cingulate gyrus	0.1568
Right inferior temporal gyrus	0.1559
Right orbital inferior frontal gyrus	0.1475
Right supplementary motor area	0.1436
Left supplementary motor area	0.1434
Right middle frontal gyrus	0.1425
Left superior temporal gyrus	0.1413
Left inferior temporal gyrus	0.1405

According to the principle of feature vector centrality, the higher the value of feature vector centrality of a particular node, the more important the node is. Therefore, in this experiment, we selected the left middle cingulate region as the core region, as it had the highest EC value.

### Statistical Analysis Results Before and After CLM Successive Failure Attacks

In this experiment, we used the clustering coefficient and global efficiency as two graph theory indicators for study and analysis.

**FIGURE 2:**
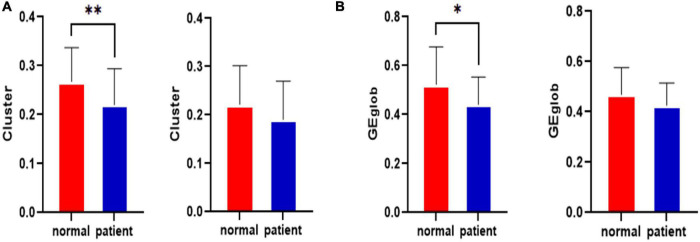
Statistical clustering coefficient and global efficiency results for the left middle cingulate gyrus before and after the attacks in the healthy control group and the JME group. **(A)** Clustering coefficient results. **(B)** Global efficiency results. *p < 0.05, ^**^p < 0.01, FDR-corrected.

The statistical results for the clustering coefficients are shown in Figure 2A. The left side of Figure 2A shows the clustering coefficients before the CLM attack for the healthy control group and the JME group. The right side of Figure 2A shows the clustering coefficients after the CLM attack for the healthy control group and the JME group. The results showed that the cluster values of the brain networks were significantly different between groups before the CLM successive failure attacks (*p* < 0.01, FDR-corrected). The cluster values of the brain networks were not significantly different after the CLM attack between the healthy control group and the JME group.

The statistical results regarding global efficiency are shown in Figure 2B. The left side of Figure 2B shows the global efficiency values before the CLM attack for the healthy control group and the JME group. The right side of Figure 2B contains the global efficiency results obtained after the CLM attack for the healthy control group and the JME group. The results showed that the global efficiency values of the brain networks were significantly different between groups before the CLM successive failure attacks (*p* < 0.05, FDR-corrected). The global efficiency values of brain networks were not significantly different after the CLM attacks between the healthy control group and the JME group.

### The Rich-Club Results

In this study, the rich-club organization of the brain network was studied to explore the changes in the brain network before and after the occurrence of successive failures. Two-sample *t*-tests and a variance analysis were performed on the three types of connections (rich connections, local connections, and feeder connections) before and after CLM sequential failure attacks in the healthy control group and the JME patient group.

**FIGURE 3:**
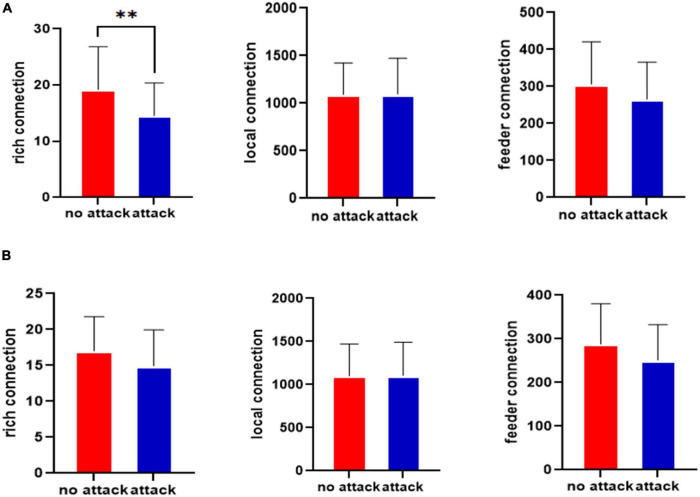
Statistical testing results for the rich-club junction of the left middle cingulate gyrus before and after the attacks in the healthy control group **(A)** and JME group **(B)**. ^**^p < 0.01, FDR-corrected.

The rich-club connections before and after the left middle cingulate gyrus that was attacked for the healthy control group and the JME group are shown in Figure 3. Figure 3A shows the rich-club junction in the attacked left middle cingulate gyrus for the healthy control group. Figure 3A shows that the rich connections in the brain network exhibited statistically significant differences before and after the CLM attacks (*p* < 0.01, FDR-corrected), while there were no significant differences in the feeder and local connections. Figure 3B shows the rich-club junction in the attacked left middle cingulate gyrus of the JME group. The results showed that the rich, feeder, and local connections were not statistically different in the brain network before and after the CLM attack (*p* > 0.05, FDR-corrected) for the JME group.

**FIGURE 4:**
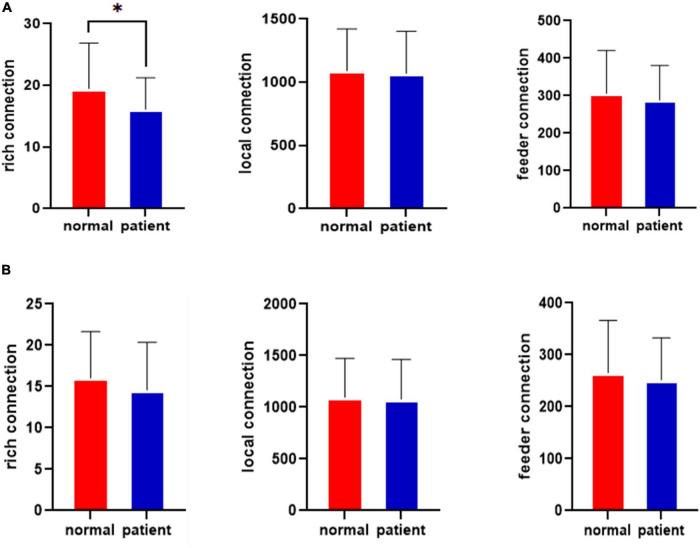
Results for the rich-club junctions in the healthy control group and the JME group. **(A)** Results for the rich-club junctions before the attacks. **(B)** Results for the rich-club junctions after the attacks. *p < 0.05, FDR-corrected.

The rich-club connections before the left cingulate gyrus attack for the healthy control and JME groups are shown in Figure 4A. The results in Figure 4A show that the rich-club organization of the brain network in the JME patient group was changed from that in the healthy control group before the CLM attack. The number of rich connections was significantly reduced (*p* < 0.05, FDR-corrected), while the feeder connections and local connections were not significantly different. The rich-club connections after attacking the left middle cingulate gyrus in the healthy control group and the JME group are shown in Figure 4B. The results showed that there were no significant differences in the rich, feeder, and local connections after the CLM attacks in the healthy control group and the JME group (*p* > 0.05, FDR-corrected).

The variance analysis results for the rich clubs before the attack in the healthy control group and JME group show that there were fewer rich connections in the brain networks of the patients with JME (*p* <0.05) than in those of the healthy controls before the CLM attack. The feeder and local connections were not significantly different.

The variance analysis results for the rich clubs before and after the attack in the healthy control group show that there were fewer rich connections in the brain networks of the healthy controls (*p* < 0.01) before the CLM attack. The feeder and local connections were not significantly different. No statistically significant differences were observed among the rich connections, feeder, or local connections after the CLM attacks for the healthy controls.

The variance analysis results for the rich clubs before and after the attack in the JME group show that the rich, feeder, and local connections in the JME group before and after the CLM attacks were not significantly different.

The variance analysis results for the rich clubs after the attacks in the healthy control group and the JME group show that the rich, feeder, and local connections after the CLM attacks were not significantly different between the healthy control group and the JME group. These results were consistent with those of the rich-club single-sample *t*-test.

Additionally, to better understand the relationships between the damage to the rich-club topological structure and patients’ diseases, we performed a correlation study between the rich connections and patient disease durations.

**FIGURE 5:**
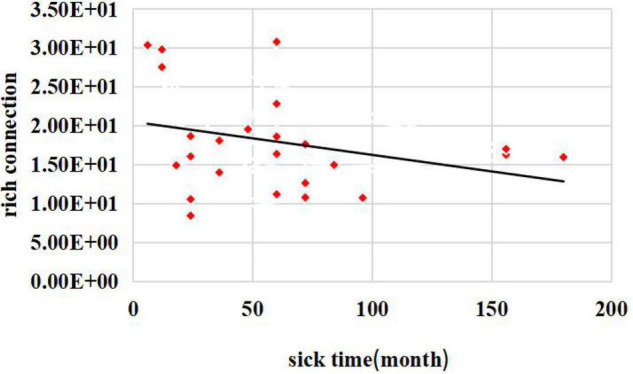
Relationship between disease duration and the number of rich connections.

The relationship between disease duration and the number of rich connections is shown in Figure 5. The abscissa represents the disease duration for patients with JME, and the ordinate denotes the patient’s rich connection weights. The results showed that there was a negative correlation between the rich connection weights and the patients’ disease durations. This result showed that the weight values of rich connections in the brain network decreased as the illness duration increased.

## Discussion

In this paper, we examined JME disorders in terms of network modularity and network topological organization. The EC algorithm was used to calculate the EC values of 90 brain regions. The left middle cingulate gyrus was determined as a core brain region, and its role in JME disease was studied. This method was more feasible than the previous method of selecting core nodes according to their degree centrality (Yu et al., 2018).

Previous studies of the cingulate gyrus have shown that atrophy of the right caudal anterior cingulate cortex (cACC) may contribute to reduced performance in functional tasks such as the stroop and auditory consonant trigrams (ACT), although this may be only one node of a broad brain network involved in these cognitive processes (Merkley et al., 2013). Functional connectivity between the cingulate gyrus and other brain regions is abnormal in cortical areas that facilitate motor function and sensory-motor integration (Clemens et al., 2013). The anterior cingulate cortex (ACC) is known to be involved in functions such as emotion, pain, and cognitive control (Parvizi et al., 2013). The cingulate motor areas project to the spinal cord and red nucleus and have premotor functions, while the nociceptive area is engaged in both response selection and cognitively demanding information processing. Cingulate epilepsy syndrome provides important support from experimental animal and human functional imaging studies for the role of anterior cingulate cortex in movement, affect, and social behaviors. Excessive cingulate activity in patients with seizures confirmed in the anterior cingulate cortex *via* subdural electrode recordings can impair consciousness, alter affective state and expression, and influence skeletomotor and autonomic activity. The above findings suggest that, in JME, the cingulate gyrus is involved in both cognitive and motor activity in the brain network. Abnormalities in this brain region cause abnormalities in both cognitive and motor activity in the brain network. This is consistent with the clinical appearance of cognitive and motor deficits in JME and suggests that the cingulate gyrus has an important role in the study of JME. This corroborates the previous results on feature vector centrality. Both in conjunction with the feature vector centrality algorithm and in relation to previous studies of the middle cingulate gyrus, our selected left middle cingulate brain region does play an important role in the brain network.

To study the influence of cingulate gyrus malfunction on the brain network, the clustering coefficient and global efficiency of the brain network were calculated, and the results were statistically analyzed. We found that the clustering coefficient and global efficiency results before and after successive CLM failure attacks exhibited significant differences. The clustering coefficient represented an important parameter regarding the degree of node connections. The observed significant reduction in the clustering coefficient indicated that the node connections related to the left middle cingulate gyrus were blocked after successive failure attacks, which led to a significant decrease in the clustering coefficient results. Global efficiency represents the global information transmission capability of the brain network. After successive failure attacks, the transmitted and received information that passed through the middle cingulate gyrus in the brain network was interrupted. This phenomenon led to information transmission capability changes.

To investigate the effect of the left cingulate gyrus on the network topology, we used CLM successive failure attacks on the central brain region to study the rich-club organization differences in the brain networks of healthy controls and patients with JME before and after the attacks. First, we calculated the rich-club connections of the healthy controls and patients with JME before executing CLM successive failure attacks. The results showed a statistically significant difference between the numbers of rich connections in the group of patients with JME and the healthy control group. The number of rich connections was significantly reduced (*p* < 0.05, FDR-corrected). The feeder connection and local connection results showed no significant difference between the two groups. The above results suggest that there were fewer rich-club connections in the brain networks of patients with JME than in those of the healthy controls before CLM successively failed to attack the left middle cingulate gyrus. The most significant results were for the rich connections, which indicated that the rich-club tissue in the brain networks of the patients was damaged. A comparison between the rich-club results of the healthy subjects and patients with JME before and after CLM successive failure attacks showed that only the rich connections in the healthy controls exhibited statistically significant differences before and after the attacks (*p* < 0.01, FDR-corrected). There was no significant difference between the feeder connections and local connections. No significant differences were observed among the rich connections, local connections, and feeder connections in the patients with JME before and after successive CLM failure attacks on the left middle cingulate gyrus. This indicated that left middle cingulate gyrus lesions were already present in the group of patients with JME, so there were no significant differences before and after successive CLM failure attacks. The rich-club organization results of healthy subjects and patients with JME after successive CLM failure attacks showed no significant differences in terms of the rich connections, feeder connections, and local connections (*p* > 0.05, FDR-corrected) after successive CLM failure attacks. This means that the results changed from a significant difference regarding the rich connections before the attack to no significant difference in the healthy controls after successive CLM failure attacks on the left middle cingulate gyrus. This suggested that the rich-club connections in the healthy control brain networks after successive failure attacks were similar to the rich-club connections in the patients with JME. The hub regions were damaged in the patients with JME relative to those of normal subjects, and the left middle cingulate gyrus abnormalities might be related to the physiological mechanism of juvenile myoclonic seizures. Damage to the left middle cingulate gyrus in healthy controls was likely to induce disease in healthy subjects. At the same time, to better understand the relationship between the impairment of the rich-club topological structure and disease, we performed a correlation study between rich connections and patients’ disease durations. We found that there was a negative correlation between the rich connection weights and patients’ illness durations. This result showed that the weight values of rich connections in the brain network decreased as the duration of illness increased.

These results suggested that the left middle cingulate gyrus in patients with JME was impaired compared with that in normal subjects; this may be related to the physiological mechanism of adolescent clonic seizures. The rich-club organization was made up of three types of connections. Rich connections in the rich club referred to the connections between rich nodes, which were located in the central position of brain network. Rich connections played a key role in achieving whole-brain neural signal reception and communication between brain regions, which affected many structural and functional properties of the network, including its topology, path efficiency, and load distribution (Csigi et al., 2017). The left middle cingulate fell within the category of rich nodes. Therefore, for healthy controls before the successively CLM failure attacks, the central brain region functioned normally in terms of the rich-club connections and graph theory indicators, and the rich connection weight values were normal. In the JME group, the left middle cingulate gyrus was damaged, and the rich connection weight values were outside of the normal range. On this basis, the rich-club connections in the healthy control group and the group of patients with JME were significantly different before the successive CLM failures. The left middle cingulate gyrus lesions were simulated after successive CLM failure attacks on the core brain region. After the lesions, the neural signals passing through the rich nodes and the communication information between the functional network regions were greatly reduced. This caused more information that should be transmitted through this road in the brain network to be blocked. Then, the communication information of the whole brain was greatly reduced in terms of a reduction in the number of rich connections. The topology and function connections of the brain network and the graph theory indicators were reorganized (Zhu et al., 2016). Therefore, the rich connections exhibited significant differences for the healthy controls before and after the CLM attacks. The impacts of the left middle cingulate gyrus on the local connections and feeder connections were smaller. No significant differences were observed between the local connections and feeder connections. Similarly, the “rich node” of the midbrain network of the left middle cingulate node was diseased before the CLM attacks in each patient with JMEt, which resulted in the reorganization of the topological structure of the rich-club connections of the brain network, and the weight values of the rich connections were reduced. Therefore, the CLM sequential failure attack was carried out again. There was no significant change in the weight values of the rich connections. No significant differences were observed between the rich connections before and after the CLM attacks in the group of patients with JME. The middle cingulate gyrus had little influence on the local connections and feeder connections. There were no significant differences between the local connections and feeder connections. After the CLM attacks, left middle cingulate gyrus legions were found in both the healthy control group and the group of patients with JME, and the changes in the left middle cingulate gyrus legions of the brain networks of the two groups were relatively consistent. The results showed that the rich-club connections in the two brain networks changed from exhibiting a significant difference before the attacks to presenting no significant difference. This suggested that damage to the left middle cingulate gyrus in healthy controls is likely to induce disease in healthy subjects. Research papers have shown a link between damaged brain connectivity and disease (Ansari et al., 2019).

It is worth noting that the cingulate gyrus could also integrate other neural networks and had close connections between its interneurons (Bagla and Skidmore, 2011; Unnwongse et al., 2012). The middle cingulate gyrus is a part of the cingulate gyrus and plays a role in communication between the anterior and posterior cingulate gyri. Therefore, the function of the left middle cingulate gyrus might be similar to that of the cingulate gyrus, and it may play an important role in communicating with other neural networks. Studies on the cingulate gyrus have confirmed that the middle cingulate gyrus is also known as the cingulate motor area (CMA). The middle cingulate gyrus is located below the pre-supplementary motor area (SMA) between the vertical commissure anterior (VCA) and vertical commissure posterior (VCP) lines (McConachie and King, 1997), and it is another cortical area that is associated with the motor area (Vogt et al., 1992). The middle cingulate gyrus, which lies below the presubjective motor area (pre-SMA), had projections to the anterior part of the subjective motor area, the dorsolateral prefrontal cortex, the primary motor cortex, the inferior parietal cortex, the reticular formation and motor thalamus, the red nucleus, and the spinal cord. The middle cingulate gyrus exhibited three characteristics with respect to information processing; i.e., it had a unique way of processing emotional, sensory, motor, and cognitive information. It could also integrate inputs from multiple sources, including motivation formations, error predictions, and cognitive and affective network reappearances. It also affected activity in other brain regions, such as the regulation of motor, cognitive, endocrine, and visceral responses (Devinsky et al., 1995; Bagla and Skidmore, 2011; Unnwongse et al., 2012). From an anatomical point of view, the cingulate has a unique emotional, sensory, motor, and cognitive information process (Bagla and Skidmore, 2011; Unnwongse et al., 2012). At the functional level of the brain, the middle cingulate might be involved in the ability to communicate with other neural networks of the brain and the ability to modulate executive functions. Combined with the role of the middle cingulate gyrus regarding the anatomy and functional connectivity of the brain network, it is known that left middle cingulate gyrus lesions can, indeed, cause abnormalities in neural network communication within the brain network and abnormalities in terms of motor area function, which would lead to physiological abnormalities at the motor level in patients. This is consistent with the descriptions of myoclonic seizures, atonic seizures, generalized tonic-clonic seizures, cognitive impairment, and motor impairment in the clinical presentation of JME. This result suggested that left middle cingulate gyrus lesions had the potential to cause abnormalities in the functional interactions among brain networks, which, in turn, led to motor impairment in patients with JME.

Studies of patients with middle cingulate epilepsy have suggested that middle cingulate epilepsy might be associated with paramotor movements and might manifest clinically as generalized tonic-clonic seizures (GTCSs), tonic seizures or paroxysmal seizures (Devinsky et al., 1995). Lim et al. reported that the main manifestations of middle cingulate epilepsy are spastic seizures (Lim et al., 2004). It has also been shown that the clinical manifestations of middle cingulate epilepsy are spastic seizures (Schrader et al., 2009). A recent study has reported protean manifestations of epileptic seizures that have been ascribed to the cingulate gyrus (Lim et al., 2004). These studies suggested that the left cingulate gyrus plays a role in the disease not only by regulating the communication between brain networks and other neural networks and regulating executive functions but also by affecting motor area function. The left middle cingulate gyrus lesions exhibited spastic seizures, which was consistent with the descriptions of the physiological phenomena of JME clinical manifestations (myoclonic seizures, motor seizures, generalized tonic-clonic seizures, cognitive impairment, and motor deficits). It was further demonstrated that the abnormalities of the left middle cingulate gyrus were, indeed, related to the functional interactions among brain networks and motor disorders from a pathological perspective. Therefore, the findings of our study showed that abnormalities in the left middle cingulate gyrus might lead to brain network topological reorganization and motor deficits. Our findings suggested that the left middle cingulate gyrus is a key brain region in patients with JME and is strongly associated with the pathogenesis of JME. However, the present experiment also has limitations. Brain mapping was performed in the present experiment with the AAL-90 without the cerebellum. According to the function of the cerebellum, abnormalities in this region are also associated with motor deficits. Therefore, the absence of the cerebellar template in the AAL-90 brain atlas could have affected the results of our experiment. We will address this limitation in subsequent experiments.

## Data Availability Statement

The raw data supporting the conclusions of this article will be made available by the authors, without undue reservation.

## Ethics Statement

The studies involving human participants were reviewed and approved by the Epilepsy Center of Lanzhou University Second Hospital. Written informed consent to participate in this study was provided by the participants’ legal guardian/next of kin.

## Author Contributions

MK, HL, and GL designed the experiment and revised the manuscript. MK and HL wrote the manuscript. GL recorded and collected the data. HL performed the data analysis. All authors contributed to the article and approved the submitted version.

## Conflict of Interest

The authors declare that the research was conducted in the absence of any commercial or financial relationships that could be construed as a potential conflict of interest.

## Publisher’s Note

All claims expressed in this article are solely those of the authors and do not necessarily represent those of their affiliated organizations, or those of the publisher, the editors and the reviewers. Any product that may be evaluated in this article, or claim that may be made by its manufacturer, is not guaranteed or endorsed by the publisher.

## Footnotes

1
https://github.com/sandywang/GRETNA
